# A Feasibility Study on Timber Moisture Monitoring Using Piezoceramic Transducer-Enabled Active Sensing

**DOI:** 10.3390/s18093100

**Published:** 2018-09-14

**Authors:** Jicheng Zhang, Yong Li, Yongshui Huang, Jinwei Jiang, Siu-Chun M. Ho

**Affiliations:** 1 School of Urban Construction, Yangtze University, Jingzhou 434023, China; 100995@yangtzeu.edu.cn (J.Z.); 201672327@yangtzeu.edu.cn (Y.L.); 201672324@yangtzeu.edu.cn (Y.H.); 2 Department of Mechanical Engineering, University of Houston, Houston, TX 77204, USA

**Keywords:** moisture-content detection, timber, piezoceramic transducer, active sensing method, wavelet packet-based energy analysis

## Abstract

In recent years, the piezoceramic transducer-enabled active sensing technique has been extensively applied to structural damage detection and health monitoring, in civil engineering. Being abundant and renewable, timber has been widely used as a building material in many countries. However, one of the more challenging applications of timber, in construction, is the potential damage caused by moisture. Increased moisture may cause easier warping of timber components and encourage corrosion of integrated metal members, on top of potentially causing rot and decay. However, despite numerous efforts to inspect and monitor the moisture content of timber, there lacks a method that can provide truly real time, quantitative, and non-invasive measurement of timber moisture. Thus, the research presented in this paper investigated the feasibility of moisture-content monitoring using an active sensing approach, as enabled by a pair of the Lead Zirconate Titanate (PZT) transducers bonded on the surface of a timber specimen. Using a pair of transducers in an active sensing scheme, one patch generated a designed stress wave, while another patch received the signal. While the active sensing was active, the moisture content of the timber specimen was gradually increased from 0% to 60% with 10% increments. The material properties of the timber correspondingly changed under varying timber moisture content, resulting in a measurable differential in stress wave attenuation rates among the different specimens used. The experimental results indicated that the received signal energy and the moisture content of the timber specimens show a parabolic relationship. Finally, the feasibility and reliability of the presented method, for monitoring timber moisture content, are discussed.

## 1. Introduction

Timber, which is a ubiquitous natural resource, is used widely across many countries as a building material [1,2]. Timber is an inhomogeneous, anisotropic organic material whose mechanical properties are affected by several factors, such as its specific cellular structure, as well as the physical, and chemical conditions of the surrounding environment. Moisture content (MC) is one of the key influencing factors. Different moisture content can cause variations in timber properties, such as in the strength, the stiffness, and the physical volume. Moisture may even initiate decay or encourage the growth of fungi, which reduces the mechanical strength of the timber [3]. Therefore, given the vast number of timber-based structures around the globe, the investigation for determining a reliable detection method of moisture content in timber and wooden structures is of great significance.

Traditionally, wood moisture content is estimated by weighing the wood [4]. By comparing the weight of the wet wood to that of its dried (i.e., using an oven) condition, the moisture mass can be estimated. Recently, with the rapid development in structural health monitoring methods [5,6,7,8,9,10,11] and damage detection technologies [12,13], the identification of moisture content in timber and wooden structures has attracted much attention. For instance, Brischke et al. [14,15] and Fredriksson et al. [16,17] determined the moisture content of wood by measuring the change of electrical resistance due to the presence of absorbed moisture. Subsequently, Yamamoto et al. [18] used a modified confocal laser scanning microscope (CLSM) system to observe in-situ microcracks on wooden surfaces, and measure the change in resistivity, to obtain information about moisture content. Casans et al. [19] proposed an analog circuit, for high resistance measurement of fiber materials, to estimate the moisture content of wood, based on the measurements of resistance in the fiber material and its relationship with moisture content. Fredriksson et al. [20] and Björngrim et al. [21] applied conductivity measurement methods to evaluate the moisture content of wooden structures. Similarly, radio frequency techniques [22,23,24,25,26], capacitance measurements [27,28,29,30,31,32], fiber optics [33,34,35], and *X*-ray techniques [36,37,38,39,40] were all used to assess the moisture content of wood, and provide new ideas for nondestructive evaluation of wood moisture contents. Recently, Rodriguez–Abad et al. [41] and Reci et al. [42] employed the wave signal, based on the ultrasonic method, and the Ground Penetrating Radar (GPR) method to estimate the moisture content of wood, respectively, and they discovered noticeable changes in measurement data when the wood moisture content changed. However, most of the above-mentioned methods are qualitative in nature. In addition, due to the need for manually driven external excitation, the above methods are not suitable for real time monitoring.

Recently, methods for structural damage detection [43,44,45,46] and health monitoring [47,48,49,50,51,52,53] have been developed to identify the damage status and health of structures, in real time. In particular, the active sensing method is based on the piezoelectric effect of piezoelectric materials through which health monitoring and damage detection in structures are achieved. Piezoceramic transducers based on Lead Zirconate Titanate (PZT) are widely used in the active sensing method due to their several advantages, such as rapid response [54], energy harvesting capacity [55,56], low cost, ease of implementation [57,58,59,60,61], and the dual effects of both sensing and actuating [62,63,64]. Wand et al. [65] and Roh [66] proposed the active sensing monitoring technique to diagnose the damage of composite plates, by embedding multiple piezoelectric patches into a composite structure. Subsequently, this technique is widely applied in the damage detection and structural health monitoring in civil and mechanical engineering, such as for damage detection in a pipeline system [67,68,69], timber structures [70], monitoring of bolt looseness [71,72,73,74], damage detection in concrete structures [75,76,77], monitoring of soil water content [78], soil freeze–thaw process [79], bond slip detection of composite concrete structures [80,81], and debonding in adhesively-bonded structures [82,83]. However, the study of moisture-content monitoring in timber or wooden structures, using PZT transducer-enabled active sensing approach, has not been reported.

In this research, we propose the usage of a PZT transducer-enabled active sensing method to monitor timber moisture content and carry out experimental feasibility studies by using timber specimens. For each specimen, one PZT patch served as the actuator and another served as the sensor. The actuator generates stress waves that propagate through the structure and are received by the sensors. As the propagation characteristics of the stress wave are sensitive to timber moisture content, the stress wave energy will change correspondingly. A wavelet packet-based energy index was applied to evaluate the moisture content. The results indicated that this method can estimate the moisture content in timber structure quantitatively and accurately.

## 2. Principles

### 2.1. Active Sensing Method

**Figure 1:**
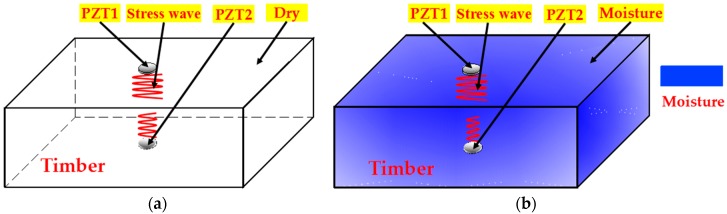
Stress-wave-based active sensing approach to monitor timber moisture content. (**a**) Dry timber (relatively stronger wave detected by PZT2); (**b**) timber with higher moisture-content level (relatively weaker wave detected by PZT1).

The active sensing method as enabled by PZTs was used to estimate the moisture content in timber samples. In this method, one PZT patch (“PZT1”) generates a stress wave that propagates across the sample and is received by another PZT patch (“PZT2”). Both patches are bonded to the top and bottom surfaces of the sample. Changes in the sample lead to changes in the received signal. Figure 1 depicts the application of the method to a timber sample in both dry and wet conditions. A designed, directional stress wave containing frequency components from 100 Hz to 500 kHz was generated by PZT1 and received by PZT2. Due to the change in the timber moisture content, the material properties of the timber will change and result in a corresponding change in the stress wave attenuation ratio in timber. To quantify the timber moisture content, a wavelet packet-based energy approach was used (Section 2.2).

### 2.2. Wavelet Packet-Based Energy Approach

The wavelet packet analysis approach has several desirable characteristics, such as high time-frequency resolution, and it can effectively decompose and analyze frequency signals across much of the frequency spectrum. Wavelet packet analysis can also be used to obtain data insight, in both time and frequency domains. The wavelet packet-based energy approach is often used in structural analysis to compute the energy of received signals [84,85]. In this investigation, a wavelet packet-based energy analysis was used to compute the received wave signal energy under different moisture content in timber specimens, which is given as follows:

First, the original signal *S* received by the PZT sensor was decomposed by an *n-*level wavelet packet decomposition into 2*^n^* signal subsets with different frequency bands. The signal subset *X_j_*, where *j* was the frequency band (*j* = 1, 2, …, $2^{n}$), could be expressed as,

$$X_{j}=\left[X_{j,1},X_{j,2},\cdots ,X_{j,m}\right]$$

where *m* was the data sampling of the decomposed signal subset.

Second, the energy of the signal subset $E_{i,j}$, could be defined as

$$E_{i,j}={∥X_{j}∥}^{2}=X_{j,1}^{2}+X_{j,2}^{2}+\cdots +X_{j,m}^{2}$$

where *i* was the *i*th measurement.

The energy vector of the signal at the *i*th measurement could be given as,

$$E_{i}=\left[E_{i,1},E_{i,2},\cdots ,E_{i,2^{n}}\right]$$

Finally, based on the definition of the energy vector *E_i_*, the total energy *E* of the received original signal at the *i*th measurement could be computed as,

$$E=\overset{2^{n}}{\underset{j=1}{\sum }}E_{i,j}$$

In this paper, the received wave signal energy under different moisture content in timber specimens was computed via the wavelet packet-based energy method.

## 3. Experimental Setup

### 3.1. Timber Specimen

**Figure 2:**
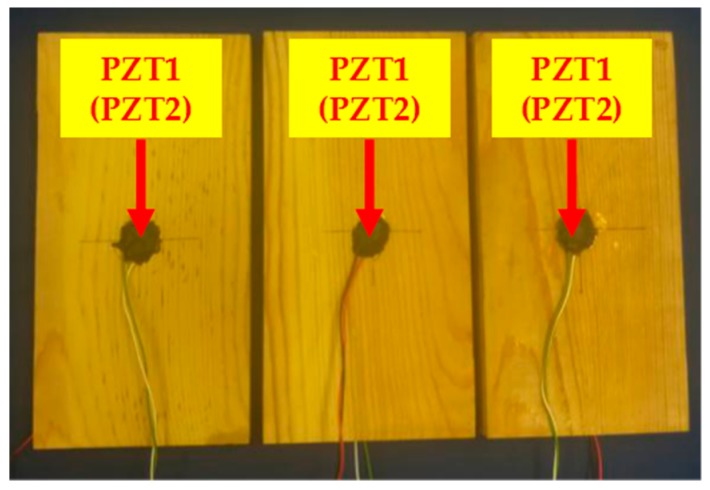
Timber specimens. (PZT2 is at the same position, on the back side). From left to right: Specimen 1, Specimen 2, Specimen 3.

In this experiment, a total of three timber specimens with the same dimensions (200 mm × 100 mm × 20 mm) were fabricated using the same pine wood from North America. For each test specimen, a pair of PZT disks (10 mm diameter, 0.2 mm thick, purchased from Beijing Ultrasonic) were mounted onto predetermined positions, using epoxy (Loctite Heavy Duty 5 min epoxy) (Figure 2). In this research, the type of the PZT sensors used was PZT-5H. The PZT sensor was a sandwiched structure, with two electrode layers and one layer of PZT material.

Moisture content (MC) has different definitions in the literature; for the purposes of this paper, the MC of the timber specimens in this study was defined as:

$$MC=\frac{m_{water}}{m_{dry}}\times 100\%=\frac{m_{wet}-m_{dry}}{m_{dry}}\times 100\%$$

where *MC* was the moisture content of the timber specimen, *m_water_* was the mass of water within the timber specimen, *m_dry_* was the mass of the dry timber specimen, *m_wet_* was the total mass of the wet timber specimen. In this study, *m_dry_* of the timber specimen was determined by the Chinese national standard (GB/T 1931–2009) [86]. According to the standard protocol, the timber specimens were placed in an oven and baked at a temperature of (103 ± 2) °C for 8 h, then the mass of the timber specimens was weighed and recorded on an electronic scale. Subsequently, the selected specimens were weighed every 2 h. The *m_dry_* of the timber specimens were determined when the difference between the two most-recent measurements did not exceed 0.5%.

### 3.2. Experimental Setup

**Figure 3:**
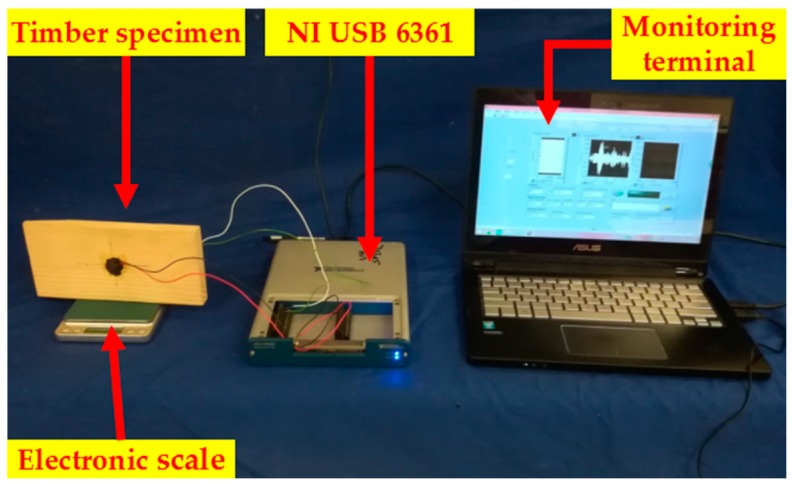
The experimental setup.

**Figure 4:**
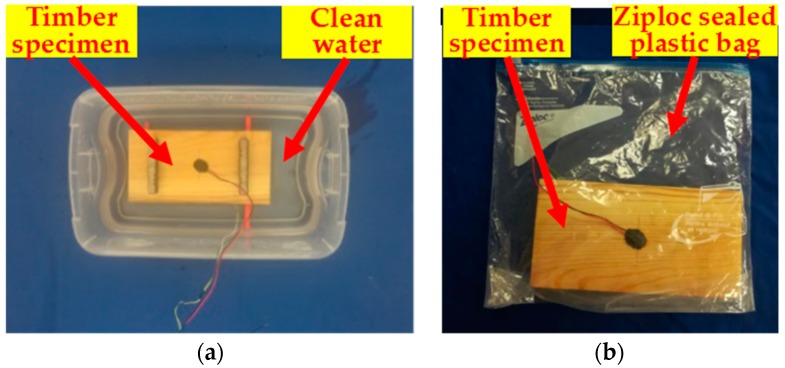
Moisture exposure. (**a**) Immersed in water; (**b**) contained in Ziploc sealed plastic bag.

The experimental setup consisted of a data acquisition system (National Instruments (NI) USB-6361), timber specimens, an electronic scale and a monitoring terminal (a laptop), as shown in Figure 3. During the test, an electronic scale was used (Accuracy: 0.01 g) to measure the mass of the wet timber specimen, after it was immersed into clean water until the MC reached the designed value (60%) (Figure 4a). The moisture content of the timber specimen gradually increased from 0% to 60% with 10% increments. When the MC of the timber specimen reached the designed value, the timber specimen was placed into a Ziploc sealed plastic bag for more than 12 h (Figure 4b) to ensure the uniformity of moisture in the timber. At every 10% MC increase, a swept sine excitation signal was input to the PZT actuator to transmit a stress wave towards the other end of the specimen. PZT patches were layered in epoxy for waterproofing. Experimental details of swept sine wave signals are shown in Table 1.

## 4. Results and Discussions

**Figure 5:**
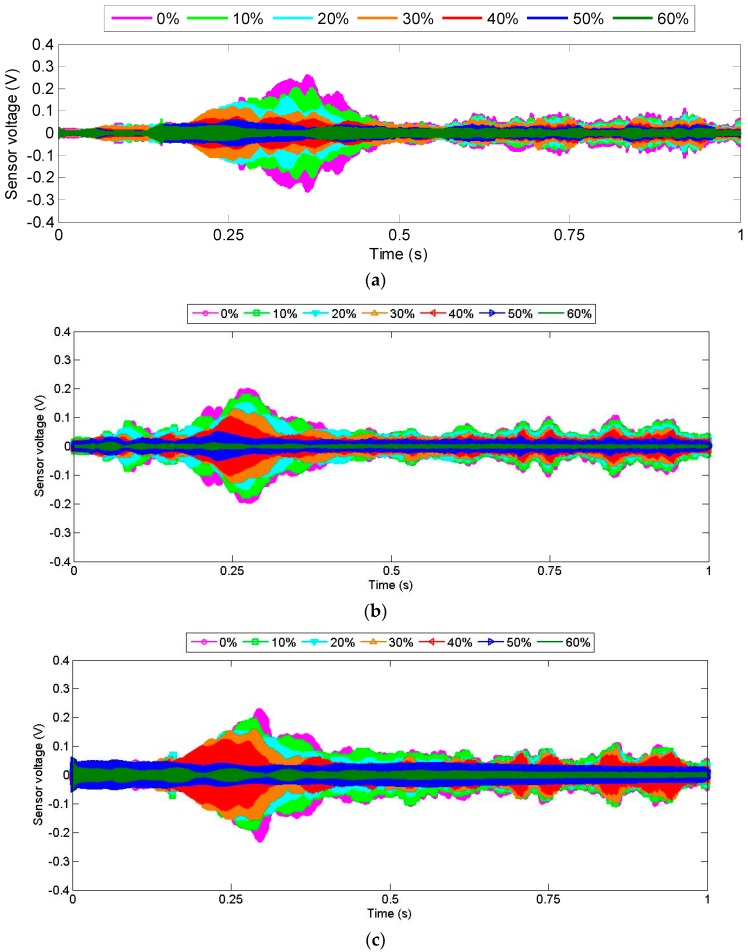
Sensor signal response for timber specimens. (**a**) Specimen 1; (**b**) specimen 2; (**c**) specimen 3.

The signals received by the PZT sensors, in the time domain, under different moisture-content levels (0%, 10%, 20%, 30%, 40%, 50%, and 60%) are shown in Figure 5. The data indicated that the amplitude of the signal received by the PZT sensor decreased when the moisture content in the timber specimen increased. As water provides an attenuating effect for stress waves, such an inverse correlation might be expected. On the other hand, despite sharing the same overall trend, each specimen still exhibited unique characteristics in the received signal, perhaps due to minor non-uniformities among specimens, including epoxy thickness and electrode welding.

**Figure 6:**
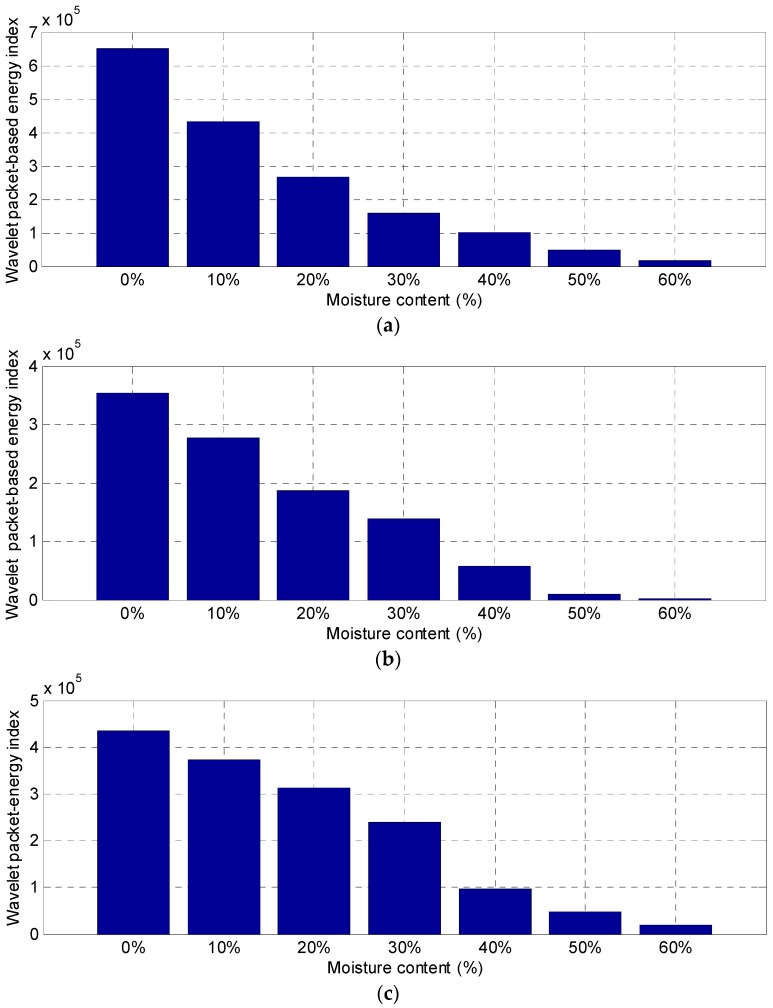
Energy indices of timber specimen with different moisture content. (**a**) Specimen 1; (**b**) specimen 2; (**c**) specimen 3.

In order to analyze the changes in stress wave response, the energy of the received signal was estimated using the wavelet packet-based energy method (Figure 6). The observed trend in the wavelet packet-based energy of the three timber specimens indicated a decrease in signal energy with the increase of the moisture content. Furthermore, the correlation between signal energy and MC suggested a parabolic relationship. However, there was a slight difference in the energy value of the three timber specimens for the same moisture-content levels. The reason for the slight discrepancies could be the natural inhomogeneity of timber, which significantly affects the stress wave propagation.

The experimental results showed that piezoceramic transducers hold great promise for use in the monitoring of moisture content of wooden structures through an active sensing method. On the other hand, certain challenges should be addressed prior to practical applications. First, although the moisture content of timber and wooden structures could be estimated directly from the time domain response and processed by the wavelet packet-based energy index, these methods could not be used to identify moisture-content distribution in timber and wooden structures. Second, the research did not consider certain aspects, such as the temperature, the species of wood, the geometry of the samples, as well as any boundary conditions, defects, and properties of the epoxy. Controlling for these parameters may lead to further insight into the results.

## 5. Conclusions

This paper demonstrated, for the first time, the use of PZT-enabled active sensing techniques to monitor the moisture content of timber specimens in real time. The amplitude of the wave signal received by PZT sensor decreased with the increase of moisture content in the timber specimens. The energy of the received signals, computed by using the wavelet packet-based energy approach, could be employed to quantitatively evaluate the change in moisture content of the timber specimens. Additionally, a parabolic relationship was found between the stress wave signal energy and the moisture content of the timber specimens. The experimental results revealed that the active sensing technique, based on PZT transducers, was effective and sensitive to be able to monitor the moisture content of the timber specimens, in real time. Future work in this area could include an investigation of the sensitivity and reliability of the method, the feasibility of the proposed method for quantitatively monitoring the moisture content on a larger scale, and in-service timber structures and structural elements. However, the investigations also need to consider additional influencing factors, such as the bonding layer, temperature, humidity, boundary conditions, and the microstructure of the wood.

## Figures and Tables

**Table 1:** Experimental details of the swept sine wave signal.

Start Frequency (Hz)	Stop Frequency (kHz)	Amplitude (V)	Period (s)
100	500	10	1
